# Regret on Choice of Colorectal Cancer Screening Modality Was Associated with Poorer Screening Compliance: A 4-Year Prospective Cohort Study

**DOI:** 10.1371/journal.pone.0125782

**Published:** 2015-04-14

**Authors:** Martin C. S. Wong, Jessica Y. L. Ching, Victor C. W. Chan, Renee Bruggemann, Thomas Y. T. Lam, Arthur K. C. Luk, Justin C. Y. Wu, Francis K. L. Chan, Joseph J. Y. Sung

**Affiliations:** 1 Institute of Digestive Disease, Faculty of Medicine, Chinese University of Hong Kong, Shatin, Hong Kong, HKSAR, China; 2 School of Public Health and Primary Care, Faculty of Medicine, Chinese University of Hong Kong, Shatin, Hong Kong, HKSAR, China; East Tennessee State University Quillen College of Medicine, UNITED STATES

## Abstract

**Purpose:**

Very few studies examined the issue of regret on choosing colorectal cancer (CRC) screening tests. We evaluated the determinants of regret and tested the hypothesis that regret over screening choices was associated with poorer screening compliance.

**Methods:**

A bowel cancer screening centre invited all Hong Kong citizens aged 50-70 years who were asymptomatic of CRC to participate in free-of-charge screening programmes. Upon attendance they attended health seminars on CRC and its screening, and were offered an option to choose yearly faecal immunochemical test (FIT) for up to four years vs. one direct colonoscopy. They were not allowed to switch the screening option after decision. A self-administered, four-item validated survey was used to assess whether they regretted over their choice (> 2 = regretful from a scale of 0 [no regret]-5 [extreme regret]). A binary logistic regression model evaluated if initial regret over their choice was associated with poorer programme compliance.

**Results:**

From 4,341 screening participants who have chosen FIT or colonoscopy, 120 (2.8%) regretted over their decision and 1,029 (23.7%) were non-compliant with the screening programme. Younger subjects and people who felt pressure when making their decision were associated with regret. People who regretted their decision were 2.189 (95% C.I. 1.361-3.521, p = 0.001) times more likely to be non-compliant with the programme.

**Conclusions:**

This study is the first to show that regret over the initial CRC screening choice was associated with later non-compliance. Screening participants who expressed regret over their choice should receive additional reminders to improve their programmatic compliance.

## Introduction

Colorectal cancer (CRC) is the second most common cancer in women and third in men worldwide [1]. It accounted for 10% of all malignancies and 8% of all cancer deaths globally, leading to a substantial public health burden [1]. The recent decade witnessed a two- to three-fold rise in the incidence of CRC in the Asia-Pacific countries, including China, Japan, Korea, Singapore and Hong Kong [2]—which is now comparable with the incidence figure in Western nations.

CRC screening is effective to reduce mortality as randomized trials have shown that Faecal Occult Blood Tests (FOBT) and colonoscopy can reduce cancer deaths by up to 33% [3–5] and 68% [6–7], respectively. Therefore, guidelines from the US Preventive Task Force [8] and the Asia Pacific consensus statements [9] have recommended FOBT and colonoscopy as suitable primary screening tools for population-based CRC screening. Nevertheless, the uptake rate of CRC screening programme and its persistent compliance remained low in many nations including Asian countries [10–13]. This has been attributed to poor knowledge of CRC screening, as well as various psychological and health system barriers [14, 15].

A previous telephone survey conducted in Hong Kong [16] based on the Health Belief Model (HBM) evaluated a few variables pertaining to HBM which were associated with CRC testing, namely perceived severity of CRC; perceived health and psychological barriers to CRC testing; and perceived access barriers to testing. This clearly highlights the importance of psychological perception as a key factor which could modify screening uptake. Decisional regret is one of the important psychological constructs which might adversely affect healthcare behavior. It has been defined as the decisional consequence characterized by a feeling of self-blame or disappointment with respect to a choice that failed to produce a desired outcome [17]. It could derive from underlying psychosocial stress or dysfunctional communications with healthcare providers when decisions are being reached [18]. A recent meta-analysis concluded that interventions which consist of a regret component could influence the likelihood of patients’ intention being translated into behavior, like exercise, weight loss, condom use and screening uptake [19]. However, the regret component of the meta-analysis studied was anticipated regret, and it is currently unknown whether the experience of perceiving “decisional regret” on the choice CRC screening tools could influence persistent compliance with CRC screening.This study aims to evaluate the factors associated with the perception of regret in a CRC screening practice. In addition, we tested the *a priori* hypothesis that regret over the choice of CRC screening methods was associated with poorer screening compliance over time.

## Materials and Methods

### Ethics Statement

This study was approved by the Clinical Research Ethics Committee of the Chinese University of Hong Kong. All study participants provided informed written consent.

The setting for this study has been described elsewhere [14, 15, 20–24]. We have previously conducted a study in the same setting evaluating the factors associated with programme compliance over time [23], as well as whether informed choice is associated with better compliance. Briefly, a bowel cancer screening centre was established in Hong Kong in 2008. It invited free CRC screening for eligible Hong Kong residents aged 50 to 70 years who were asymptomatic of CRC via media invitations. This community centre provides education and CRC screening to a large population of Hong Kong, and is accessible to all Hong Kong residents. We collected data based on screening recruitment between 2008 and 2012.

### Study Design

The centre prospectively recruited self-referred screening participants aged 50 to 70 years for CRC screening who could register via telephone, fax, email, or walk-in.

#### Participant recruitment

The eligibility criteria for this study were (i) age 50 to 70 years; (ii) absence of existing or previous symptoms suggestive of CRC such as haematochezia, malena, anorexia or change in bowel habit in the past 4 weeks, or weight loss of greater than 5 kg in the past 6 months; and (iii) not having received any CRC screening tests in the past 5 years. Exclusion criteria included personal history of CRC, colonic adenoma, diverticular disease, inflammatory bowel disease, prosthetic heart valve or vascular graft surgery. Participants with medical conditions which were contraindications for colonoscopy, like cardiopulmonary insufficiency and the use of double antiplatelets, were also excluded. The eligibility of each participant and the exclusion criteria were checked by trained staff in the centre.

#### Survey instrument and study logistics

Registered participants were invited to fill in a self-administered questionnaire, including information on their age, gender, family history of CRC, smoking status, drinking habit, past medical history and long-term medication use. Meanwhile, centre staff checked for the completeness of questionnaires and trained volunteers assisted survey completion for illiterate participants by reading the questions word-by-word. All participants were then offered an educational session using a standard video followed by health talks by trained educators. The video included information on the epidemiology and natural history of colorectal cancer, risk factors, clinical features of this condition, importance of regular screening, and procedures of Faecal Immunochemical Test (FIT) and colonoscopy. The potential risks and benefits of FIT and colonoscopy were further explained by bowel cancer educators in the centre. All educators were trained by a team of gastroenterologists, family physicians and public health professionals prior to the programme. The talks were delivered in a standardized manner with both FIT and colonoscopy being presented in a non-preferential manner. Each session lasted for approximately one and a half hour, and was limited to a maximum audience size of 30. Interaction among participants was discouraged, and they were given a choice between yearly FIT (Hemosure Inc, El Monte, CA) for up to five years or a direct colonoscopy for CRC screening. They were requested to make their choice within 30 minutes after the talks, which resembled real-life screening practices. All participants who have chosen their preferred screening tests were not allowed to change their choice. They completed a survey to measure their level of regret immediately afterwards.

#### Outcome Variables

A scale measuring the degree of regret was devised by a panel of academic professors, gastroenterologists, family physicians, public health experts and social workers. The decisional regret here referred to having decided to undergo a certain CRC screening test (i.e., FIT or colonoscopy). It consists of 4 survey items, namely “I made the right decision”; “I regret my decision”; “I will make the same decision if I choose again” and “My decision is harmful to me”. A five-point Likert scale ranging from 1 to 5 was devised for each survey item, including “strongly agree”, “agree”, “neutral”, “disagree” and “strongly disagree”. Two items were negatively worded to ensure that the respondents read each question carefully as a safeguard against response bias. These survey items were pilot-tested in 20 screening participants for comprehensibility and wordings were modified by the panel according to their comments. All the responses of these four items were averaged with the possible score ranging from 1 (no regret) to 5 (extremely regret). To measure compliance with the screening programme, subjects who chose yearly FIT were followed up for four years whilst those chosen colonoscopy were followed up until the scheduled endoscopy appointment. Non-compliance was defined as no return of faecal test samples for at least one year, or non-attendance to colonoscopy. The study participants who had positive faecal tests yet defaulted colonoscopy follow-up were also regarded as non-compliant.

### Statistical Analyses

All data were entered into a predesigned database with logistic checking using Microsoft Access, and analyzed using SPSS software, version 18.0 (Chicago, Illinois). The proportion of participants who reported “strongly agree” or “agree” for the four regret survey items were compared between the FIT and colonoscopy groups. The overall regret scores were presented according to age; gender; educational level; perception of the necessity for people aged >50 years to undergo CRC screening; prior CRC screening examination; their need for more information on CRC screening; knowledge scores on CRC; whether the participants changed their mind on the preferred screening option before and after the health talks; the degree of pressure felt whilst making the decision; and the scores on satisfaction of the screening programme. To evaluate the factors associated with experiencing regret, univariate analysis was conducted consecutively for each covariate mentioned above. All covariates were included into a binary logistic regression model if the initial p value is less than 0.10 in the univariate analysis. To test for the association between regret and compliance, a multivariate regression model was constructed with compliance as the outcome, controlling for all the covariates. All the variables selected in the multivariate regression analysis were detected for the presence of interactions. Since the cut-off point of 2 for the scale measuring regret was arbitrary, two more cut-off points at 2.5 and 3, respectively, were used to detect if the findings were similar. As part of sensitivity analysis, the regret score was converted to a continuous scale and the same analyses were repeated to explore for any heterogeneity. All p-values <0.05 in the multivariate regression analysis were regarded as statistically significant.

## Results

### Participant characteristics

From 4,341 screening participants who visited the centre, 1,975 chose FIT and 2,366 chose colonoscopy (**Table 1**). Their average age was 57.7 (SD 4.9) years and 41.9% were male subjects. Among them, subjects choosing colonoscopy were younger; had higher educational level; more were married; greater proportions worked full-time; more perceived the necessity for people aged older than 50 years to undergo CRC screening; had higher household income; and more had family history of CRC (**Table 1**).

**Table 1:** Participant Characteristics (N = 4,341).

	Colonoscopy (n = 1,975)	Faecal Tests (n = 2,366)	p value
***Gender*, *n (%)***			
Male	840 (42.5)	977 (41.3)	0.41
Female	1135 (57.5)	1389 (58.7)	
***Age*, *n (%)***			
50–54	714 (36.2)	754 (31.9)	<0.001
55–59	675 (34.2)	744 (31.4)	
60–64	413 (20.9)	539 (22.8)	
65–70	173 (8.8)	329 (13.9)	
***Education level*, *n (%)***			
Primary or below	528 (26.7)	752 (31.8)	0.001
Secondary	1136 (57.5)	1302 (55.0)	
Tertiary or above	306 (15.5)	307 (13.0)	
***Marital status*, *n (%)***			
Married	1711 (86.6)	1978 (83.6)	0.005
Single/ divorced/ widowed/ other	264 (13.4)	388 (16.4)	
***Occupational status*, *n (%)***			
Full time	761 (38.5)	751 (31.7)	<0.001
Part time or retired	615 (31.1)	819 (34.6)	
Housewife and others	599 (30.3)	796 (33.6)	
***Necessity for people aged >50 years to undergo CRC screening*, *n (%)***			
Very high or high	1729 (87.6)	1880 (79.50)	<0.001
Low or very low	47 (2.4)	112 (4.7)	
Not sure	198 (10.0)	374 (15.8)	
***Monthly household income ($US)***			
<1289$	599 (30.3)	757 (32.0)	<0.001
1289–2579$	579 (29.3)	676 (28.6)	
2579–3868$	286 (14.5)	294 (12.4)	
3868–5158$	134 (6.8)	131 (5.5)	
>5158$	146 (7.4)	129 (5.5)	
Refused to answer	231 (11.7)	379 (16.0)	
***Body Mass Index (kg/m*** ^***2***^ ***)***			
<23	889 (46.5)	996 (46.1)	0.807
≧23	1024 (53.5)	1165 (53.9)	
***Family history of CRC***			
Nil	1043 (52.8)	1473 (62.3)	<0.001
First degree relatives	289 (14.6)	231 (9.8)	
Second degree relatives	260 (13.2)	285 (12.0)	
Others	383 (19.4)	377 (15.9)	
***Smoking***			
Non-smoker/ ex-smoker	1881 (95.2)	2242 (94.8)	0.47
Current smoker	94 (4.8)	124 (5.2)	

CRC: Colorectal Cancer.

The study was conducted from 2008–2012 in Hong Kong.

### Responses on the regret survey after choice of a CRC screening modality

The vast majority of the participants agreed or strongly agreed that they made a right decision (94.4%); they did not regret about the decision (93.0%); they would make the same decision if they could choose again (94.8%); and the decision made was not harmful (91.6%) (**Table 2**). The average regret score among all subjects was 1.79; and 2.8% had a regret score ≥ 2. When compared with subjects chosen FIT, higher proportions of subjects chosen colonoscopy perceived that they had made a right decision (96.0% vs. 93.1%, p<0.001), and would made the same decision if they could choose again (96.9% vs. 93.3%, p<0.001). The average regret score was 1.82 and 1.74 in the FIT and colonoscopy group, respectively. In univariate analysis, female gender, lower educational level, doubt about the necessity for people aged >50 years to undergo CRC screening, perceived need for more information about CRC, lower CRC knowledge score, changing of screening choice after health talks, feeling of pressure whilst making decision, and lower satisfaction of the screening programme was associated with higher levels of regret (**Table 3**).

**Table 2:** Responses of the study participants on the regret survey.

Regret	Colonoscopy (n = 1,975)	Faecal Tests (n = 2,366)	p value
*I made the right decision*, *n (%)*			
Strongly agree or agree	1896 (96.0)	2200 (93.1)	<0.001
Neutral	60 (3)	133 (5.6)	
Strongly disagree or disagree	19 (1.0)	29 (1.2)	
*I do not regret about my decision*, *n (%)*			
Strongly agree or agree	1856 (94.3)	2181 (92.6)	0.079
Neutral	64 (3.3)	92 (3.9)	
Strongly disagree or disagree	49 (2.5)	82 (3.5)	
*I will make the same decision if I choose again*, *n (%)*			
Strongly agree or agree	1911 (96.9)	2205 (93.3)	<0.001
Neutral	43 (2.2)	106 (4.5)	
Strongly disagree or disagree	19 (1.0)	53 (2.2)	
*My decision is not harmful to me*, *n (%)*			
Strongly agree or agree	1800 (91.5)	2175 (92.2)	0.674
Neutral	83 (4.2)	90 (3.8)	
Strongly disagree or disagree	85 (4.3)	94 (4.0)	

The study was conducted from 2008–2012 in Hong Kong.

**Table 3:** Regret scores among study participants chosen colonoscopy and faecal tests.

	Colonoscopy		Faecal Tests	
	Regret Score*	p value	Regret Score*	p value
***Gender*, *n (%)***				
Male	1.69 (n = 836)	<0.001	1.79 (n = 968)	0.001
Female	1.78 (n = 1126)		1.87 (n = 1380)	
***Age*, *n (%)***				
50–54	1.75 (n = 712)	0.496	1.85 (n = 750)	0.367
55–59	1.76 (n = 670)		1.83 (n = 737)	
60–64	1.72 (n = 408)		1.85 (n = 533)	
65–70	1.71 (n = 172)		1.79 (n = 328)	
***Education level*, *n (%)***				
Primary or below	1.82 (n = 519)	0.001	1.86 (n = 745)	0.187
Secondary	1.72 (n = 1132)		1.84 (n = 1292)	
Tertiary or above	1.70 (n = 306)		1.79 (n = 306)	
***Necessity for people aged >50 years to undergo CRC screening*, *n (%)***				
Very high or high	1.73 (n = 1722)	<0.001	1.82 (n = 1866)	0.024
Low or very low	1.87 (n = 47)		1.83 (n = 112)	
Not sure	1.87 (n = 192)		1.90 (n = 370)	
***Previous bowel examination*, *n (%)***				
No	1.75 (n = 1851)	0.394	1.83 (n = 2249)	0.146
Yes	1.70 (n = 111)		1.91 (n = 99)	
***Need for more information*, *n (%)***				
Strongly agree or agree	2.09 (n = 27)	0.001	2.30 (n = 56)	<0.001
Neutral	1.74 (n = 1771)		1.82 (n = 2094)	
Strongly disagree or disagree	1.69 (n = 161)		1.89 (n = 189)	
CRC knowledge score**, *n (%)*				
<5	1.85 (n = 1526)	0.067	1.78 (n = 1133)	<0.001
≧5	1.81 (822)		1.69 (n = 829)	
***Changed mind after health talk*, *n (%)***				
Did not change mind	1.72 (n = 1475)	<0.001	1.81 (n = 843)	0.154
Changed mind	1.82 (n = 487)		1.85 (n = 1505)	
***Feeling pressure whilst making the decision*, *n (%)***				
0–2	1.71 (n = 361)	<0.001	1.86 (n = 387)	<0.001
3 or 4	1.87 (n = 290)		2.00 (n = 285)	
5 or 6	2.01 (n = 334)		2.06 (n = 382)	
7 to 10	2.05 (n = 128)		2.09 (n = 106)	
Overall satisfaction score*, *n (%)*				
1.0–1.4	1.34 (n = 753)	<0.001	1.42 (n = 737)	<0.001
1.5–1.9	1.71 (n = 149)		1.83 (n = 166)	
2.0–2.4	2.02 (n = 1019)		2.02 (n = 1348)	
2.5–2.9	2.37 (n = 28)		2.29 (n = 40)	
3.0–3.4	2.33 (n = 12)		2.50 (n = 53)	

CRC: Colorectal Cancer

The study was conducted from 2008–2012 in Hong Kong.

*Rated on a scale from 1 through 5. *The lower the score*, *the more favorable the response*

** *Rated on a scale from 1 through 9*. *The higher the score*, *the better the knowledge*

### Factors associated with experiencing regret

From binary logistic regression analysis, younger age (AOR [adjusted odds ratio] = 0.64 to 0.72 for those aged 60–70 years; referent: 50–54 years); uncertainty about the necessity to screen people aged ≥ 50 years (AOR = 1.54, 95% C.I. 1.12–2.12, p = 0.009), and feeling pressure whilst making decision (AOR = 2.29 to 2.63) were significantly associated with higher levels of regret (**Table 4**).

**Table 4:** Factors associated with experiencing regret (score ≥ 2.0 out of 5.0) after choice of CRC screening option.

	Colonoscopy				Faecal Tests				All participants			
Patient Characteristics	Crude OR (95% C.I)	p value	Adjusted OR (95% C.I.)	p value	Crude OR (95% C.I)	p value	Adjusted OR (95% C.I.)	p value	Crude OR (95% C.I)	p value	Adjusted OR (95% C.I.)	p value
*Gender*, *n (%)*												
Male	Referent		Referent		Referent		Referent		Referent		Referent	
Female	1.28 (1.069–1.533)	0.007	1.01 (0.768–1.339)	0.919	1.22 (1.033–1.450)	0.02	1.23 (0.931–1.619)	0.146	1.25 (1.109–1.418)	<0.001	1.14 (0.941–1.386)	0.179
*Age*, *n (%)*												
50–54	Referent	0.082	Referent	0.008	Referent	0.127			Referent	0.047	Referent	0.01
55–59	0.93 (0.753–1.154)	0.518	0.90 (0.657–1.236)	0.517	0.89 (0.721–1.102)	0.288			0.91 (0.787–1.062)	0.242	0.95 (0.758–1.201)	0.69
60–64	0.77 (0.606–0.989)	0.04	0.69 (0.477–1.007)	0.054	0.93 (0.734–1.166)	0.51			0.87 (0.735–1.026)	0.098	0.72 (0.554–0.926)	**0.011**
65–70	0.72 (0.512–0.999)	0.05	0.43 (0.249–0.726)	**0.002**	0.73 (0.556–0.947)	0.018			0.75 (0.614–0.926)	0.007	0.64 (0.456–0.908)	**0.012**
*Education level*, *n (%)*												
Primary or below	Referent	0.871			Referent	0.54			Referent	0.361		
Secondary	0.95 (0.770–1.171)	0.628			0.99 (0.821–1.194)	0.916			0.96 (0.831–1.097)	0.516		
Tertiary or above	0.89 (0.669–1.180)	0.415			0.83 (0.630–1.087)	0.174			0.84 (0.688–1.018)	0.075		
*Necessity for people aged >50 years to undergo screening*, *n (%)*												
Very high or high	Referent	0.005	Referent	0.017	Referent	0.045	Referent	0.708	Referent	<0.001	Referent	0.021
Low or very low	1.24 (0.686–2.233)	0.479	1.28 (0.495–3.290)	0.613	0.99 (0.669–1.465)	0.96	1.20 (0.573–2.497)	0.632	1.12 (0.810–1.553)	0.49	1.38 (0.768–2.473)	0.282
Not sure	1.68 (1.226–2.301)	0.001	2.21 (1.276–3.836)	**0.005**	1.35 (1.064–1.715)	0.013	1.16 (0.776–1.728)	0.474	1.521 (1.259–1.838)	<0.001	1.54 (1.115–2.119)	**0.009**
*Previous bowel examination*, *n (%)*												
No	Referent				Referent				Referent			
Yes	1.16 (0.788–1.697)	0.458			1.37 (0.887–2.127)	0.154			1.04 (0.782–1.380)	0.792		
*Need for more information*, *n (%)*												
Strongly agree or agree	Referent		Referent	0.299	Referent	<0.001	Referent	0.020	Referent	<0.001	Referent	0.007
Neutral	0.356 (0.143–0.887)	0.027	0.58 (0.186–1.837)	0.358	0.19 (0.082–0.450)	<0.001	0.35 (0.124–1.009)	0.052	0.24 (0.130–0.446)	<0.001	0.47 (0.220–1.009)	0.053
Strongly disagree or disagree	0.336 (0.129–0.876)	0.026	0.83 (0.237–2.926)	0.776	0.28 (0.113–0.685)	0.005	0.61 (0.192–1.907)	0.391	0.28 (0.148–0.541)	<0.001	0.76 (0.329–1.749)	0.517
CRC knowledge score*, n (%)												
<5	Referent		Referent		Referent		Referent		Referent		Referent	
≧5	0.74 (0.619–0.888)	0.001	0.77 (0.587–1.018)	0.067	0.83 (0.693–0.983)	0.031	0.86 (0.651–1.140)	0.300	0.77 (0.678–0.870)	<0.001	0.83 (0.681–1.003)	0.054
*Changed mind after health talk*, *n (%)*												
Did not change mind	Referent		Referent		Referent		Referent		Referent		Referent	
Changed mind	1.35 (1.097–1.667)	0.005	1.01 (0.743–1.371)	0.954	1.00 (0.842–1.193)	0.98	1.00 (0.750–1.342)	0.982	1.25 (1.107–1.414)	<0.001	1.13 (0.933–1.368)	0.211
*Feeling pressure whilst making the decision*, *n (%)*												
0 to 2	Referent		Referent	<0.001	Referent		Referent	<0.001	Referent	<0.001	Referent	<0.001
3 or 4	2.49 (1.785–3.460)	<0.001	2.54 (1.808–3.568)	**<0.001**	2.24 (1.558–3.220)	<0.001	2.23 (1.545–3.212)	**<0.001**	2.31 (1.815–2.948)	<0.001	2.29 (1.789–2.923)	**<0.001**
5 or 6	3.85 (2.739–5.413)	<0.001	3.84 (2.695–5.471)	**<0.001**	2.01 (1.452–2.778)	<0.001	1.88 (1.351–2.613)	**<0.001**	2.77 (2.189–3.493)	<0.001	2.63 (2.068–3.334)	**<0.001**
7 to 10	3.48 (2.171–5.581)	<0.001	3.18 (1.959–5.150)	**<0.001**	1.70 (1.034–2.783)	0.036	1.66 (1.011–2.741)	**0.045**	2.44 (1.733–3.426)	<0.001	2.29 (1.619–3.228)	**<0.001**

OR: Odds Ratio; CRC: Colorectal Cancer

The study was conducted from 2008–2012 in Hong Kong.

* Rated on a scale from 1 through 9. The higher the score, the better the knowledge.

### The association between regret and persistent programme compliance

A total of 1,029 (23.7%) subjects were non-compliant with the screening programme. When non-compliance with the screening programme was used as an outcome variable in a multivariate regression model, it was found that those who experienced regret were more likely to be non-compliant (AOR = 1.53, 95% C.I. 1.198–1.940, p = 0.001) (**Table 5**). The same holds true when different cutoff values (2.5 and 3.0) were used to define regret, or when the regret score was converted to a continuous scale. There exists no interaction or multi-colinearity among covariates in the binary logistic regression model, implying that the regression analyses were robust.

**Table 5:** The association between non-compliance and the experience of regret after choosing a CRC screening option.

Feeling Regret*	Crude OR (95% C.I)	p value	Adjusted OR (95% C.I.)	p value
*Cutoff* ≧*2*.*0*	1.36 (1.174–1.571)	<0.001	1.53 (1.198–1.940)	0.001
*Cutoff ≧2*.*5*	1.25 (0.981–1.581)	0.072	1.43 (1.065–1.908)	0.017
*Cutoff ≧3*.*0*	1.96 (1.342–2.848)	<0.001	2.27 (1.433–3.608)	<0.001

OR: Odds Ratio; CRC: Colorectal Cancer

The study was conducted from 2008–2012 in Hong Kong.

*The regret scale is a four-item questionnaire with a score range from1 (no regret) to 5 (extreme regret). The binary logistic regression model adjusted for participants’ age; gender; educational level; perception of the necessity for people aged >50 years to undergo CRC screening; prior CRC screening examination; their need for more information on CRC screening; knowledge scores on CRC; whether the participants changed their mind on the preferred screening option before and after the health talks; the degree of pressure felt whilst making the decision; and their scores on satisfaction of the screening programme

## Discussion

### Major findings

This study found that 2.8% of screening participants felt regret on their choice of CRC screening tests if options were offered; when translated into population-based screening services, this seemingly modest figure indeed represents a substantial number of individuals.

It also showed that younger subjects, those who were uncertain about the necessity to screen people aged ≥ 50 years, and people who felt pressure whilst making decision were more likely to experience regret. Importantly, this regret experience was found to be significantly associated with programme non-compliance.

### Implications to clinical practice

This study bears several important implications on improving screening practices in the community. Firstly, there exist a proportion of subjects who might experience regret even after thorough education and explanation i.e. informed choice. We suggest that for participants who are rather undetermined on the screening modality, physicians should be more prescriptive—preferably with decisional aids to minimize the risk of regret. In addition, this study identified the factors associated with the experience of regret. Therefore, subjects with these factors should be given closer monitoring or intervention of their screening behavior over time. These include (1). regular and more frequent reminders around the time of their next screening round; (2). The use of small media; and (3). one-to-one education which have been found effective to enhance compliance [25]. Effective reminders include both informational booklets, as well as printed and telephone messages advising screening participants that they are due (reminder) or late (recall) for screening [25]. Its effectiveness cannot be overemphasized as screening compliance has been identified as one of the most important factors influencing the programmatic performance of CRC screening programmes. Also, this is the first study which shows that regret is significantly associated with screening compliance—and signifies the importance of psychological variables on screening behavior. Whilst a previous population-based study in Hong Kong evaluated the psychological barriers of *screening uptake*, future studies should identify the association between the perceptual variables in the HBM and *persistent compliance with screening over time*.

### Relationship with existing literature and Explanation of findings

To the best of our knowledge, this is the first study which evaluated the determinants of regret and its association with screening compliance over time. Regret is a frequently experienced, fundamental emotion in decision making [25]. In the Decision and Justification Theory (DJT), two components of regret were postulated [26]. These include regret that the *outcome* contrasts poorly with the reference outcome, and that the decision *process* was made in an unjustified manner. Since the level of regret in this study was assessed before the screening outcome, it is more likely that the study participants expressing regret were indeed induced by self-blame due to bad *decision processes* [26]. Psychological theories supported that *intention-behavior inconsistency* is a central component of the decision process. Recent studies [25] showed that the transition from intention-behavior inconsistency to regret is mediated by the judged quality of the decision process, and that regret is a warning signal about failed decision making processes and their outcomes. It is unknown why younger age was associated with a higher likelihood of regret; however, subjects who were uncertain about the necessity for screening and those who felt pressure whilst making decision might have greater difficulties to achieve intention-behavior consistency—and thus higher likelihood of regret [27]. As regret is a negative emotion which dis-incentivizes a subject’s screening behavior, it is logically plausible to be associated with poorer screening compliance.

### Study Limitations

This prospective study recruited a large number of asymptomatic subjects followed-up for 4 years. Nevertheless, there are several limitations which should be addressed. Firstly, all the study participants were self-referred and critics might argue that their levels of screening compliance were higher than that of patients who were invited by their primary care physicians for CRC screening. This might have an impact on the representativeness and generalizability of the findings. Besides, the study participants were not randomized according to the screening modalities offered, although the study objectives were not focused on comparing regret or programmatic compliance between FIT and colonoscopy groups. Also, the reasons of non-compliance have not been fully explored in this study. Some cases of non-compliance may be due to factors obviously unrelated to regret, like hospital admissions and emigration. In addition, we measured “decisional regret” instead of “anticipated regret”, and this study has not adopted decision regret scales which have already been validated, such as the instrument from the Ottawa Hospital Research Institute [28]. Our findings might also be less relevant in some healthcare systems where opportunity exists for screening participants to select other tests. Screening participants with decisional regret may need more than 30 minutes typically allocated to the screening participants.

### Conclusion

This study demonstrated an association between the experience of regret and persistent compliance with the screening programme over time. Quality improvement in the process of conducting population-based CRC screening programmes should therefore be implemented to minimize regret among the screening participants and hence enhance screening compliance. Future studies should evaluate what interventional strategies could effectively reduce the likelihood of regret among screening participants.
